# Patterns of MHC-DRB1 polymorphism in a post-glacial island canid, the Newfoundland red fox (*Vulpes vulpes deletrix*), suggest balancing selection at species and population timescales

**DOI:** 10.1007/s00251-016-0907-0

**Published:** 2016-02-19

**Authors:** H. Dawn Marshall, Barbara L. Langille, Crystal A. Hann, Hugh G. Whitney

**Affiliations:** Department of Biology, Memorial University of Newfoundland, St. John’s, Newfoundland and Labrador A1B 3X9 Canada; Animal Health Division, Forestry and Agrifoods Agency, Box 7400, St. John’s, Newfoundland A1E 3Y5 Canada

**Keywords:** Red fox, Major histocompatibility complex, DRB1, Balancing selection, Tests of neutrality, Host immunogenetics

## Abstract

As the only native insular Newfoundland canid between the extinction of the wolf in the 1930s and the recent arrival of coyotes, the red fox (*Vulpes vulpes deletrix* Bangs 1898) poses interesting questions about genetic distinctiveness and the post-glacial colonization history of the island’s depauperate mammalian fauna. Here, we characterized genetic variability at the major histocompatibility complex (MHC) class II DR β1 domain (DRB1) locus in 28 red foxes from six sampling localities island-wide and compared it with mitochondrial control region (CR) diversity and DRB1 diversity in other canids. Our goals were to describe novel DRB1 alleles in a new canid population and to make inferences about the role of selection in maintaining their diversity. As in numerous studies of vertebrates, we found an order-of-magnitude higher nucleotide diversity at the DRB1 locus compared with the CR and significantly positive nonsynonymous-to-synonymous substitution ratios, indicative of selection in the distant past. Although the evidence is weaker, the Ewens-Watterson test of neutrality and the geographical distribution of variation compared with the CR suggest a role for selection over the evolutionary timescale of populations. We report the first genetic data from the DRB1 locus in the red fox and establish baseline information regarding immunogenetic variation in this island canid population which should inform continued investigations of population demography, adaptive genetic diversity, and wildlife disease in red foxes and related species.

## Introduction

The major histocompatibility complex (MHC) contains among the most polymorphic genes in the vertebrate genome (Garrigan and Hedrick 2003). Allele frequencies (Hedrick and Thomson 1983), nucleotide sequence variation (Hughes and Nei 1988), linkage disequilibrium (Klitz and Thomson 1987), and allele genealogies (Takahata 1990) of many MHC genes are thought to be largely patterned by balancing selection, although this is a paradigm which is neither fully explanatory (Garrigan and Hedrick 2003; van Oosterhout 2009) nor is the intensity of selection estimated necessarily high (Satta et al. 1994; Yasukochi and Satta 2013). Nonetheless, in many species, variation in these prominent immune system genes is associated with disease agent pathogenicity, resistance, or susceptibility (e.g., Wassom et al. 1983; Buitkamp et al. 1996; Paterson et al. 1998; Grimholt et al. 2003; McClelland et al. 2003; Biedrzycka et al. 2011; Tsai et al. 2013). As such, the role of host genetic diversity in spread of infectious disease remains an open question in disease ecology, necessitating information from a wide variety of non-model wildlife organisms (Lively et al. 2014). From both evolutionary and ecological perspectives then, MHC genes provide a window into the study of functionally relevant adaptive genetic variation.

MHC class II genes, expressed on antigen-presenting cells, recognize extracellular pathogens such as bacteria and nematodes (Hughes and Yeager 1998). The class II gene series DP, DQ, and DR encode heterodimeric proteins each with α and β chains. The DR β1 domain (DRB1) gene, especially exon 2, has been extensively studied in mammals, in part because it encodes functionally important antigen binding sites and in part because tight linkage to other class II genes allows variability of the DRB to predict variability at other loci (reviewed in Sommer 2005). For a non-model organism such as the red fox, the DRB1 exon 2 thus provides a practical starting point to investigate functional polymorphism within the MHC.

The island of Newfoundland, part of the Canadian province of Newfoundland and Labrador and North America’s easternmost land mass, is host to only 14 indigenous terrestrial mammal species in four orders, including seven in Carnivora. Most of this fauna colonized the island post-glacially, within the last ∼7000 years (South 1983). Widely distributed in North America, Eurasia, northern Africa, and Australia, the red fox (*Vulpes vulpes* L.) comprises some 40 subspecies and occupies a broad range of habitats from tundra to urban (Larivière and Pasitschniak-Arts 1996). The Newfoundland red fox (*V. vulpes deletrix* Bangs 1898), described as pale and straw-colored relative to mainland counterparts, has been considered distinct enough from neighboring mainland populations to warrant subspecies status (Dodds 1983). Newfoundland’s red fox population is of special interest because insular Newfoundland is the only endemic focus in North America of the endoparasitic nematode French heartworm (*Angiostrongylus vasorum*), the causative agent of verminous pneumonia and coagulopathy (Jeffery et al. 2004). This parasite is of concern due to the possibility of transmission to domestic canines and the potential for spread to other parts of North America (Conboy 2011). In addition to wildlife diseases, the rapidly expanding coyote population in Newfoundland (McGrath et al. 2010) may threaten this island population of red foxes.

While studies of MHC variability tend to focus on species of conservation interest that have experienced population declines (e.g., Bowen et al. 2004; Babik et al. 2005; Huchard et al. 2006), Castillo et al. (2010) investigated the relationship between MHC diversity and the spread and maintenance of disease in a widespread and plentiful species, the North American raccoon (*Procyon lotor*), thus providing a comparative model for understanding adaptive genetic variation in non-threatened populations, as advocated by Kohn et al. (2006). Similar to raccoons, red foxes in Newfoundland present an excellent model for investigation of MHC variability in a disease-susceptible population experiencing novel challenges. Additionally, comparison of diversity at functional loci such as MHC and neutral ones such as the mitochondrial control region challenges the long-held tenet of conservation genetics that a relationship exists between levels of neutral and adaptive variation (Kohn et al. 2006). For example, a study of the MHC class II DRB and DQB loci in the San Nicolas Island fox (*Urocyon littoralis dickeyi*), one of the most monomorphic sexually reproducing animal populations known, revealed remarkably high levels of MHC variation (Aguilar et al. 2004).

The family Canidae in the mammalian Order Carnivora includes the closely related *Canis* species (dogs, wolves, and coyotes) and the progressively more distantly related South American foxes (genus *Dusicyon*), red fox-like canids (genus *Vulpes*), and grey and island foxes (genus *Urocyon*). Suites of DRB1 alleles have been described in several wild canid species including island foxes (Aguilar et al. 2004), Mexican grey wolves (Hedrick et al. 2000, 2003), red wolves and coyotes (Hedrick et al. 2002), African wild dogs (Marsden et al. 2009), and arctic foxes (Ploshnitsa et al. 2011). Our objectives here were to (1) characterize DRB1 alleles in the Newfoundland population of red foxes; (2) compare patterns of variation to mitochondrial control region (CR) variation from the same localities and DRB1 sequences from other canids; and in so doing (3) use a series of tests to evaluate the evidence for balancing selection in the current generation, on a population scale, and in the distant past. Ultimately, we aim to report the first data on DRB1 diversity in Newfoundland red foxes and establish a baseline for comparison for future studies investigating host genetic components of immunity in the Newfoundland red fox.

## Materials and methods

### Study area and sample collection

**Fig. 1:**
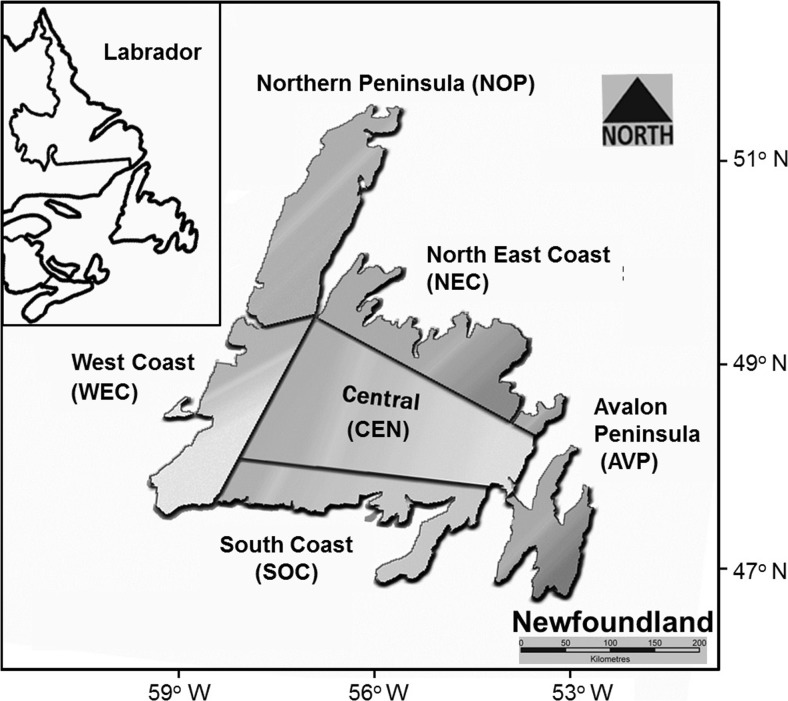
Geographic location of the six sampled island Newfoundland red fox populations (Northeast Coast, Central Newfoundland, Avalon Peninsula, West Coast, South Coast, Northern Peninsula)

Salivary glands or muscle tissue from four to five red foxes from each of six sampling regions (Avalon Peninsula, Central, Northeast Coast, Northern Peninsula, West Coast, and South Coast) covering the geographic range of insular Newfoundland (28 in total; Fig. 1 and Table 1) was collected during 2002–2004 by the Animal Health Division of the Government of Newfoundland and Labrador, as part of an insular rabies eradication program, and during 2009–2012 by wildlife trappers from the Salmonier Nature Park. Animal tissue use was approved by the Institutional Animal Care Committee of Memorial University, in accordance with Canadian Council on Animal Care guidelines.

**Table 1:** DRB1 allele and genotype frequencies among six Newfoundland island samples of red foxes

Allele	Locality						
	NOP	WEC	NEC	CEN	SOC	AVP	All
2N	10	8	10	8	10	10	56
Vuvu DRB1	2	5	1	1	3	2	14
Vuvu DRB2	3		2	4	2	3	14
Vuvu DRB3	2	1	4	1	1	2	11
Vuvu DRB4	1		1		2	1	5
Vuvu DRB5		1		1		2	4
Vuvu DRB6	2				1		3
Vuvu DRB7		1	1		1		3
Vuvu DRB8			1	1			2
Genotype							
*N*	5	4	5	4	5	5	28
2/3	1		1	1		1	4
1/1		2			1		3
1/2	2				1		3
2/4			1		1	1	3
1/5		1				1	2
1/8			1	1			2
3/6	1				1		2
3/7		1	1				2
2/5				1		1	2
1/3						1	1
3/3			1				1
2/2				1			1
4/6	1						1
4/7					1		1

**Fig. 2:**
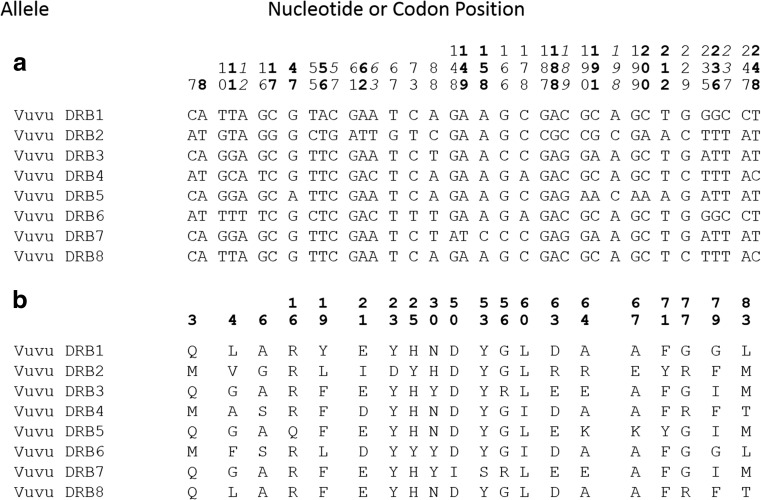
Variable sites in eight DRB1 alleles identified among 28 red foxes from six Newfoundland island localities. Alleles are designated Vuvu DRB1-8, in order of frequency of occurrence. **a** Thirty-seven variable nucleotide sites, grouped according to codon membership. Second position sites are shown in bold; third position sites are italicized. **b** Twenty variable amino acid sites, aligned with respect to the appropriate nucleotide codon

For genotype designations, a shortened version of the allele name (one for Vuvu DRB1, etc.) is used. DRB1 alleles are described in Fig. 2

*NOP* Northern Peninsula, *WEC* West Coast, *NEC* North-East Coast, *CEN* Central, *SOC* South Coast, *AVP* Avalon Peninsula

### Laboratory procedures

DNA was extracted from salivary gland tissues using a QIAamp® DNA Mini kit (Qiagen Inc., Toronto, Canada) according to the manufacturer’s instructions for tissue samples.

Exon 2 of the DRB1 locus (302 bp) was amplified with DRBIN1-F (5′-CCGTCCCCACAGCACATTTC-3′) (Aguilar et al. 2004) and HDMDRB-R (5′-CAGGCGCCCGCTGCGCTCAC-3′), modified from Aguilar et al. (2004). PCRs were performed in a final reaction volume of 25 μL containing 1× Qiagen PCR buffer, 200 μM dNTPs (New England Biolabs Inc., Whitby, Canada), 400 nM each primer, 1 U HotStar *Taq* DNA polymerase (Qiagen Inc.), and 25–200 ng DNA template. The amplification profile for DRB1 consisted of an initial denaturation for 5 min at 95 °C, followed by 14 cycles of 95 °C for 30 s, 62 °C (decreasing by 0.5 °C per cycle) for 30 s, and 72 °C for 1 min, followed by 25 cycles of 95 °C for 30 s, 55 °C for 30 s, and 72 °C for 1 min, and a final 10-min elongation at 72 °C. PCR products were purified using the QIAquick PCR Purification kit (Qiagen Inc.).

DRB1 amplicons were then cloned using the Qiagen PCR Plus Cloning kit (Qiagen Inc.) following the manufacturer’s instructions. Inserts were directly amplified from five to ten colonies for each amplicon using the M13 primers recommended by Qiagen, with reaction mixtures prepared as above but using a small portion of the bacterial colony as a template, and an amplification profile consisting of initial denaturation for 5 min at 95 °C, followed by 40 cycles of 94 °C for 30 s, 50 °C for 30 s, and 72 °C for 2 min, and a final extension at 72 °C for 10 min. PCR products were purified as above.

All PCR products were sequenced in both directions using BigDye Terminator 2.0 chemistry and electrophoresed on the ABI Prism 3130 DNA Analyzer (Applied Biosystems Inc., Foster City, USA) using Sequencing Analysis version 5.0 software. Sequence reads were assembled and edited with Sequencher version 4.9 (Gene Codes Corporation, Ann Arbor, USA).

Initial examination of MHC DRB1 exon 2 sequences identified as many as eight distinct sequences per individual fox. However, molecular cloning produces errors such as recombinant alleles, singleton mutations, and non-target DNA incorporation into the final sequence (Saitoh and Chen 2008). Following Castillo et al. (2010), to avoid such errors, distinct sequences were only considered alleles if they were not obvious recombinants of other alleles in the individual and if they differed from the other alleles in the individual by at least two nucleotide substitutions. Additionally, DRB PCR products from the 28 foxes were independently amplified and directly sequenced, and the two alleles per fox were verified only if they were observed as one of the cloned sequences inserts and could be also “phased” from the PCR product direct sequence.

### Characterization of DRB1 allele diversity in Newfoundland red foxes

Variable sites among alleles were identified and annotated with the aid of MEGA version 6 (Tamura et al. 2013), and descriptive measures of diversity (number of alleles, expected heterozygosity, nucleotide diversity, and synonymous and nonsynonymous distances), in each location and overall, were calculated using MEGA or Arlequin version 3.5 (Excoffier et al. 2007).

### Tests of selection

The following tests were performed as suggested by Garrigan and Hedrick (2003). To test for selection in the current generation, observed and expected heterozygosities (as calculated using ARLEQUIN) in each sampling location and overall were compared with chi-square tests for fit to Hardy-Weinberg expectations. Selection over the history of populations was evaluated by (1) the Ewens-Watterson test of neutrality, conducted using the software PyPop (Lancaster et al. 2003) over the entire sample of alleles—this test was also conducted for CR haplotypes from Newfoundland red foxes across the same sampling localities (Langille et al. 2014) to help dissect the effects of demography versus balancing selection on the outcome of this test, and (2) patterns of geographic variation—conventional *F*_ST_ (based on haplotype frequencies) and *d*_A_ (net number of nucleotide differences between populations; Nei and Li 1979) measures of population differentiation were calculated among each pair of sampling localities for both DRB1 data and compared to similar results from the CR data using a Mantel test, implemented in ARLEQUIN. Selection in the distant past was surveyed with (1) codon-based *Z* tests of neutrality, which test for dN–dS values different from 0 and also site-specific tests of dN–dS using the HyPhy option, as conducted in MEGA, and (2) relationships among red fox DRB1 alleles and two sets of alleles representing other canids (*Vulpes lagopus* only and *V. lagopus*, *U. littoralis*, and *Canis lupus*) accessed from GenBank were determined via neighbor-joining phylogenies of maximum composite likelihood distances and tested by 10,000 bootstrap replicates, using MEGA, as a measure of trans-species polymorphism.

## Results and discussion

Herein, we describe and characterize class II MHC DRB1 alleles in red foxes (*V. vulpes*), from the island of Newfoundland, Canada. Our goals were (1) to make inferences about the role of balancing selection over short, medium, and longer term evolutionary scales in maintaining patterns of allelic diversity at this locus in Newfoundland red foxes and (2) to introduce data regarding diversity at a functional immune system locus as a baseline for future studies in this wildlife population. We present evidence in favor of balancing selection over the medium and especially longer evolutionary timescales in this species and compare patterns of diversity in Newfoundland foxes with those observed for other canids.

### Red fox DRB1 allelic diversity

After removal of recombinants, cloned sequences that differed by only one nucleotide and any cloned sequence that could not be confirmed by direct sequencing of the PCR product, eight distinct alleles representing a 262-bp portion of the DRB1 exon 2 were identified among the 28 red foxes (deposited in GenBank with Accession numbers KU519427–KU519434) and each fox was found to contain at most two alleles, consistent with a single locus. Allele and genotype frequencies in each of the six Newfoundland red fox localities are given in Table 1. Three common alleles accounted for ∼70 % of the 56 alleles sampled: Vuvu DRB1 (25 %), Vuvu DRB2 (25 %), and Vuvu DRB3 (19.6 %). Most (23/28) foxes were heterozygotes.

**Fig. 3:**
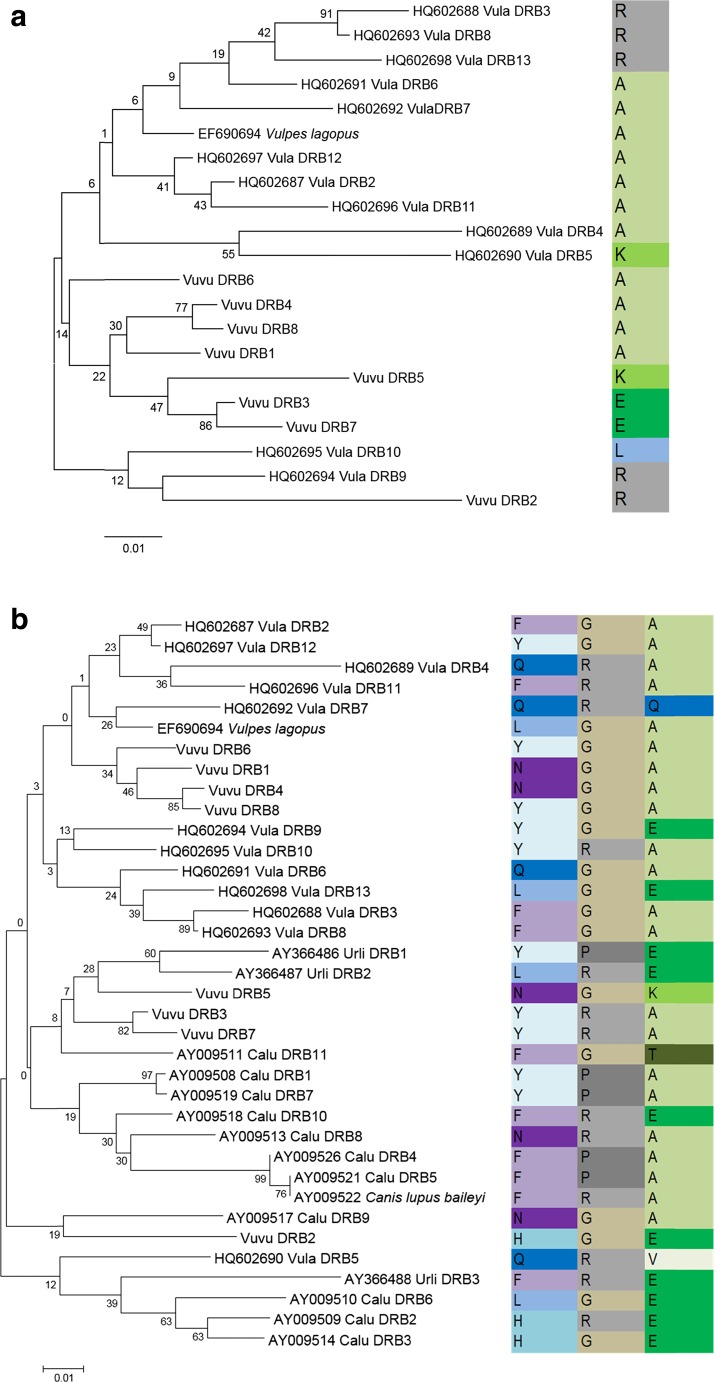
Neighbor-joining phylogenies among red fox DRB1 alleles and DRB1 alleles from other canids accessed from GenBank. Accession numbers are given with taxon name, and allele designations are as stated in the accession. Numbers above nodes are percentage support from 10,000 bootstrap replicates. The blocks to the right show the amino acid associated with each taxon at selected sites identified by HyPhy, as discussed in the text. These correspond to sites 64 (**a**) and 30, 56, and 67 (**b**) in Fig. 2b. **a** *Vulpes* only phylogeny: *Vula* = *Vulpes lagopus* and Vuvu DRB1-Vuvu DRB8 designate the *Vulpes vulpes* alleles. **b** *Vulpes*, *Urocyon*, and *Canis lupus* comprehensive phylogeny. *Urli* = *Urocyon littoralis* and *Calu* = *Canis lupus*. Due to the very large number of alleles available from *Canis* sp., *Canis lupus* was selected here to represent the lineage

Among the eight alleles, 38 nucleotide sites were variable (Fig. 2a). The vast majority of these were at first or second positions of the codon, corresponding to 20 variable amino acid sites (Fig. 2b). Alleles differed from each other by 3–26 nucleotides or 2–17 amino acids. Phylogenetic relationships among the red fox alleles are shown in Fig. 3a. While allele Vuvu DRB2 is quite distinct from the others, the only well-supported grouping of alleles are Vuvu DRB3 with Vuvu DRB7 (86 %) and Vuvu DRB4 with Vuvu DRB8 (77 %).

Nucleotide diversity for the DRB1 locus (*π* = 0.030–0.061; Table 2) is an order of magnitude higher than the CR (*π* = 0.0017–0.0062; Langille et al. 2014) and is fairly consistent across localities, although lower in the West Coast (WEC) sample. On the other hand, expected heterozygosity was in the range *H*_E_ = 0.56–0.82 and very similar to the haplotypic diversity of *h* = 0.54–0.69 for the CR (Langille et al. 2014), despite a greater number of alleles relative to sample size (eight in 2N = 56 vs seven in *N* = 159). Patterns of variability at the DRB1 locus among Newfoundland red foxes locations are uncorrelated to variability at the CR (*R*^2^ = 0.203 for nucleotide diversity and 0.076 for expected heterozygosity/haplotypic diversity; *P* > 0.05).

**Table 2:** Measures of DRB1 diversity and *Z* tests of neutrality in six Newfoundland island samples of red fox

Locality	2N	Measure of diversity						Z test of neutrality	
		*N*_A_	*H*_O_	*H*_E_	*π*	dN	dS	*Z*	*P*
NOP	10	5	1.00	0.78	0.061	0.070	0.032	2.5	0.013
WEC	8	4	0.50	0.56	0.030	0.036	0.013	2.2	0.030
NEC	10	6	0.80	0.76	0.049	0.059	0.019	2.9	0.004
CEN	8	5	0.75	0.69	0.057	0.066	0.029	2.3	0.022
SOC	10	6	0.80	0.80	0.058	0.069	0.025	3.1	0.003
AVP	10	5	1.00	0.78	0.059	0.069	0.029	2.6	0.011
All	56	8	0.82	0.82	0.054	0.064	0.026	2.7	0.007

Locality abbreviations are given in Table 1

*N* sample size, *H*_O_ observed heterozygosity, *H*_E_ expected heterozygosity, *π* nucleotide diversity, *dN* nonsynonymous substitution rate, *dS* synonymous substitution rate

### Selection at the timescales of species and populations but not in the current generation

Expected and observed measures of heterozygosity for each location and overall are presented in Table 2. There were no departures from Hardy-Weinberg expectations in any location or overall (*P* ≫ 0.05), indicating no evidence of selection in the short term. Although the small sample size investigated here may compromise power for this test, the observed and expected heterozygosities are exactly the same, so there is certainly no indication that a departure from Hardy-Weinberg equilibrium is present.

Taken together, the patterns of allele frequency and geographic distribution between the red fox CR and DRB1 alleles are consistent with selection in the medium term. The results of the Ewens-Watterson test conducted with PyPop indicate marginal (Fnd = −1.161; *P* = 0.051) evidence for departure from the expected allele frequency under neutrality for the 56 alleles at the DRB1 locus. Indeed when a simulation is done by doubling the sample size but keeping the allele distribution the same, significance is observed (*P* = 0.019). In contrast, the CR frequency distribution was quite consistent with neutrality (Fnd = −0.461; *P* = 0.396), suggesting that the result in the DRB1 locus is not due to the effects of demography. Moreover, demographic tests of expansion for the CR imply no recent expansion of this population (Langille et al. 2014).

With respect to patterns of geographic variation inferred from the DRB1 data, there was no significant differentiation among six Newfoundland localities either derived from haplotype frequencies (*F*_ST_ = 0.010; *P* = 0.317) or pairwise differences (*d*_A_ = 0.028; *P* = 0.232). Pairwise estimates of *F*_ST_ or *d*_A_ among individual localities were significant only for the WEC population compared with the NEC and Central (CEN) localities (Table 3); in fact, only the WEC and CEN samples even showed positive levels of differentiation. This patterns contrasts with results for CR in which *F*_ST_ = 0.0796 and *d*_A_ = 0.0599 among all localities were significantly different from zero (*P* ≪ 0.05), and most pairwise values were positive (Langille et al. 2014). The DRB1 and CR matrices are not correlated for either *F*_ST_ (*r* = −0.099; *P* = 0.572) or *d*_A_ (*r* = −0.236; *P* = 0.696). Lower *F*_ST_ values for the DRB1 compared with the CR may suggest uniform selection pressure across subpopulations (Garrigan and Hedrick 2003), although greater genetic drift of haploid loci may be a contributing factor.

**Table 3:** Measures of pairwise population differentiation among red fox sampling localities, inferred from DRB1 sequences

	NOP	WEC	NEC	CEN	SOC	AVP
NOP		0.095	−0.026	−0.026	−0.058	−0.047
WEC	0.107		**0.131**	**0.176**	0.022	0.070
NEC	**−**0.031	**0.145**		0.010	−0.007	−0.026
CEN	−0.034	**0.198**	0.011		0.013	−0.051
SOC	−0.070	0.023	−0.008	0.013		−0.038
AVP	−0.057	0.080	−0.031	−0.067	−0.045	

Below diagonal: *F*_ST_; above diagonal: *d*_A_. Values in bold are significant at *α* = 0.05. Locality abbreviations are as in Table 1

MHC diversity in populations facilitates the immune response to various pathogens and parasites, contributing to disease resistance at the population level. That selection has played a role in DRB1 diversity in Newfoundland red foxes at the timescale of populations does receive support from the tests of selection performed here, although certainly caution is warranted regarding the strength of this conclusion. Nonetheless, it makes sense that over the ∼7000-year period since re-colonization of Newfoundland, foxes and other species would experience novel exposure to disease and pathogens in their new environment. If particular alleles caused susceptibility or resistance to particular parasites then the frequency of those alleles may be expected to change over time. With the present data, it would be highly speculative to comment on this further with respect to particular parasites such as *A. vasorum*; however, future research effort involving documentation of association of specific alleles with disease status may be informative.

Significantly positive *Z* tests of overall dN/dS ratios were observed in Newfoundland red foxes, *V. vulpes* (Table 2). Perhaps more interesting, the HyPhy algorithm identified specific amino acid sites under significant positive selection (normalized dN–dS > 0; *P* < 0.05) in a set of *V. vulpes* and *V. lagopus* sequences (Fig. 3a) and in a more comprehensive set of canid sequences (Fig. 3b). In the former, one such site (64 in Fig. 2b) was identified. Three of the five amino acids at this site are found in alleles of both species, while the remaining two are species specific. Similarly, three sites (30, 56, and 67 in Fig. 2b; site 64 was marginally significant at *P* < 0.10) were found to be under positive selection in the inclusive data (Fig. 3b), with particular amino acid variable within species but shared between alleles of different species. For example, at site 30, all four amino acids found in the red fox are also found in alleles from the other species. Notably, three of the four sites under positive selection (30, 64, and 67) were identified by Brown et al. (1993; sites 37, 71, and 74 in the reference) as antigen binding sites. These patterns provide evidence of trans-species polymorphism at the amino acid level, consistent with *selection over the history of species*.

The neighbor-joining phylogenies among canid DRB1 alleles are shown in Fig. 3 for the two groups: all *Vulpes* (Fig. 3a) and *Vulpes*, *Urocyon*, and *C. lupus* (Fig. 3b). In the *Vulpes* phylogeny (Fig. 3a), although alleles cluster into three groups—a *lagopus* only group, a *vulpes* only group, and a mixed group—few nodes are supported by bootstrap values >50 and those that do cluster pairs of terminal branches rather than the main clusters. Similarly in the comprehensive phylogeny (Fig. 3b), there are species-specific and non-species-specific clusters but little bootstrap support at basal nodes. Thus, although the *Vulpes*-only phylogeny and the comprehensive phylogeny are consistent with trans-species polymorphism, the lack of bootstrap support prevents any strong conclusion in this respect. Nonetheless, phylogenetic relationships among *Canis* DRB1 alleles presented in earlier studies showed distinct lineages of alleles in different species supported by fairly high bootstrap estimates (Hedrick et al. 2000). This along with the evidence for shared positively selected sites found here supports balancing selection at a species timescale.

As is the case generally in mammals, patterns of DRB1 allelic diversity in Newfoundland red foxes are consistent with balancing selection in the long term, over the history of species. Evidence for this is the greater number of alleles (eight in 2N = 56 alleles cf. seven haplotypes among 159 CR sequences; Langille et al. 2014) and order of magnitude higher nucleotide divergence at the DRB1 locus in Newfoundland red foxes in comparison with the mitochondrial CR. Most of the sequence variants are associated with amino acid differences; hence, codon-based *Z* tests for departures from neutrality are strongly supportive of positive selection at a sequence level in the long term. The possibility of trans-species polymorphism in the phylogeny of DRB1 alleles among *Vulpes* and more broadly among canids suggests a role for balancing selection transcending species divergences in contributing to allelic diversity in Newfoundland red foxes.

The excess of nonsynonymous to synonymous substitutions that exists between alleles is among the most long-standing and powerful lines of evidence in favor of balancing selection at the MHC and is well documented in the peptide-binding regions of the DRB1 in a wide array of vertebrates (Garrigan and Hedrick 2003). Because this signal takes both a long time to develop and to degrade, it is difficult to make inferences about the timescale of this selection other than that it occurred in the distant past (Garrigan and Hedrick 2003). High amino acid sequence diversity of the MHC class II DRB1 polypeptide provides variation in antigen binding, providing a basis for selective pressure through the ability to resist a wider variety of parasites and pathogens (Edwards and Potts 1996). There is evidence that this is ongoing within *V. vulpes*. Indeed, the dN/dS ratio among red fox alleles is higher than among all canid alleles (∼2.4 vs ∼1.7), consistent with continuing selective pressure since species divergence, and we observe a new amino acid variant in red foxes at a site under positive selection among *Vulpes*.

### DRB1 diversity in red foxes compared with other canids

We can now compare DRB1 diversity in Newfoundland island red foxes to island populations of two other fox species: the island fox (*Uricyon littoralis*) in the Channel Islands, with a colonization history timescale of 800–16,000 years ago, and the Arctic fox (*V. lagopus*) in the Commander Islands, isolated from the mainland since the Pleistocene. Among the Channel Islands, expected heterozygosity ranges from 0 to 0.36 for a set of populations of effective populations sizes of 163–984 and levels of neutral microsatellite genetic variation on the order of 0–0.36. Notably, the San Nicolas Island population (*U. littoralis dickeyi*) showed both the lowest microsatellite and the highest DRB diversity, attributed by the authors to a severe bottleneck accompanied by strong selection (Aguilar et al. 2004). Hedrick (2004) cautioned against over-interpretation of the role of selection and suggested a combination of selective and non-selective forces to explain the patterns described by Aguilar et al. By way of contrast, in the Commander Islands, Ploshnitsa et al. (2011) observed a heterozygosity of ∼0.73 in the isolated Bering population but zero in the Mednyi population which suffered a strong bottleneck due to an outbreak of mange in the 1970s. These authors concluded that even strong balancing selection was not enough to overcome a severe bottleneck. The heterozygosity reported here (Table 2) for the Newfoundland island population of red foxes is the highest of any of these island populations, consistent with the ongoing gene flow it experiences with the mainland, as well as a much larger effective population size, and no recent bottlenecks or expansions (Langille et al. 2014).

Investigation of DRB1 alleles from *Canis*, *Vulpes*, and *Urocyon* species allows some general observations to be made. First, the dN/dS ratios are similar among the three genera (∼2.4 in *Vulpes*, ∼ 2.2 in *Canis*, and ∼1.7 in *Urocyon* [only three alleles available]). Notably, the dN measures are virtually identical among taxa, and the differences in ratios are explained by dS differences. This suggests that over the long term, there may be similar levels of selective pressure in each of these taxa. Second, while species or genera tend to group together loosely phylogenetically, the inclusion of all genera illuminates the tendency of alleles to be shared past species and even genus barriers, although this is not well supported by bootstrap analysis, probably due to the length of the sequence analyzed. We also note that unlike in *Canis*, deeper divergences within the *Vulpes* only phylogeny are not well supported by bootstrap values. This may be due to longer divergences times among species in the two genera.

## Conclusions and future directions

The signature of positive and balancing selection in red foxes in Newfoundland is consistent with the known functional significance of the genes of the MHC and particularly the DRB1 in numerous studies across vertebrates. Species which exhibit low or no detectable polymorphism in MHC genes might suffer from increased susceptibility to disease, whether enzootic or epizootic (Hedrick, 1994). Here, we also provide evidence that this may be not just in the long term but over the history of the population, as may be expected when establishing a new environment. Whether the new variants observed here are unique to this population remains to be seen.

As suggested by Castillo et al. (2010) for raccoons, the available DRB1 locus data will also allow continued spatial and temporal analysis of the immunogenetic response of red foxes to infection and the influences of diseases on molecular evolution of this important class of loci and about the role of selection and exposure to parasites in the maintenance of genetic diversity of the immune system. There is no evidence for a relationship between parasitic exposure and DRB1 diversity or particular alleles in the Newfoundland population of red foxes, nor of selection in the current generation, establishing a baseline for monitoring future genetic changes in this potentially vulnerable species. As noted by Sommer (2005), the MHC class II region regions are closely linked in mammals, so the pattern observed for the DRB1 locus should be a good indicator of the genetic variation in other class II genes. Going forward, our long-term goals are to broaden our examination to include other genes, other species, and particular references to individuals of specific disease status.
